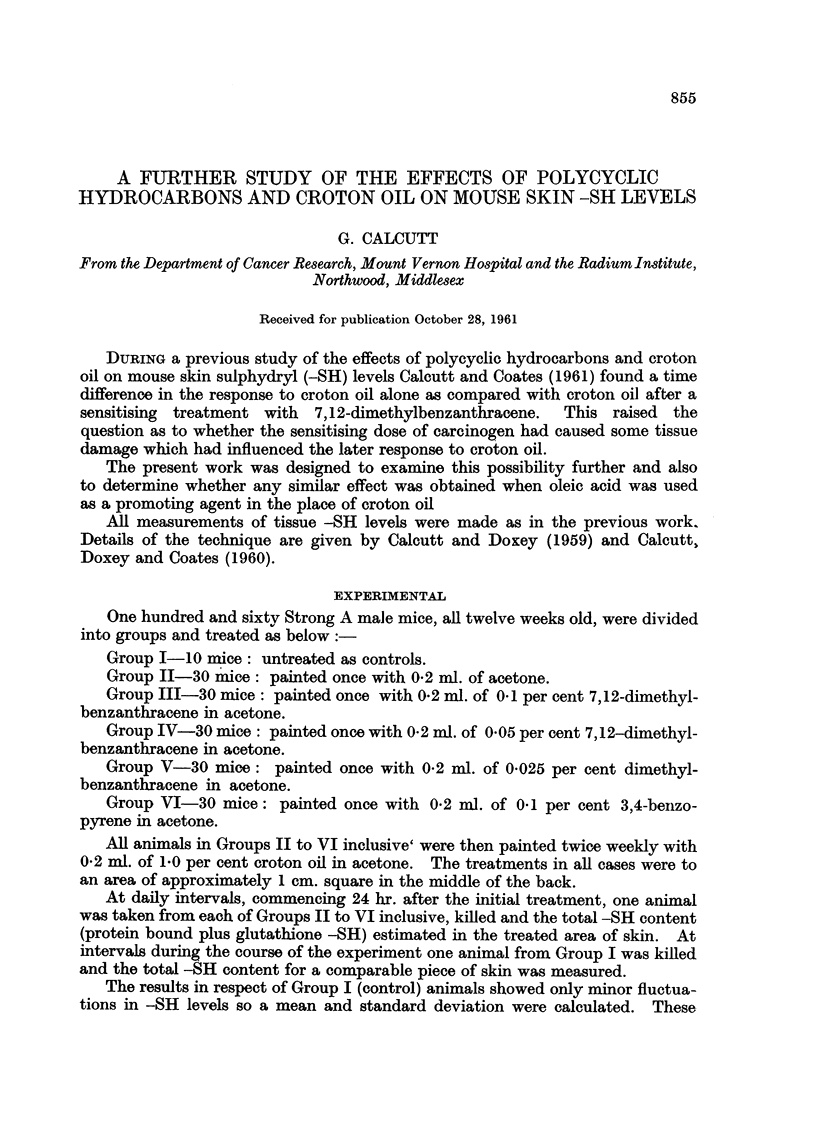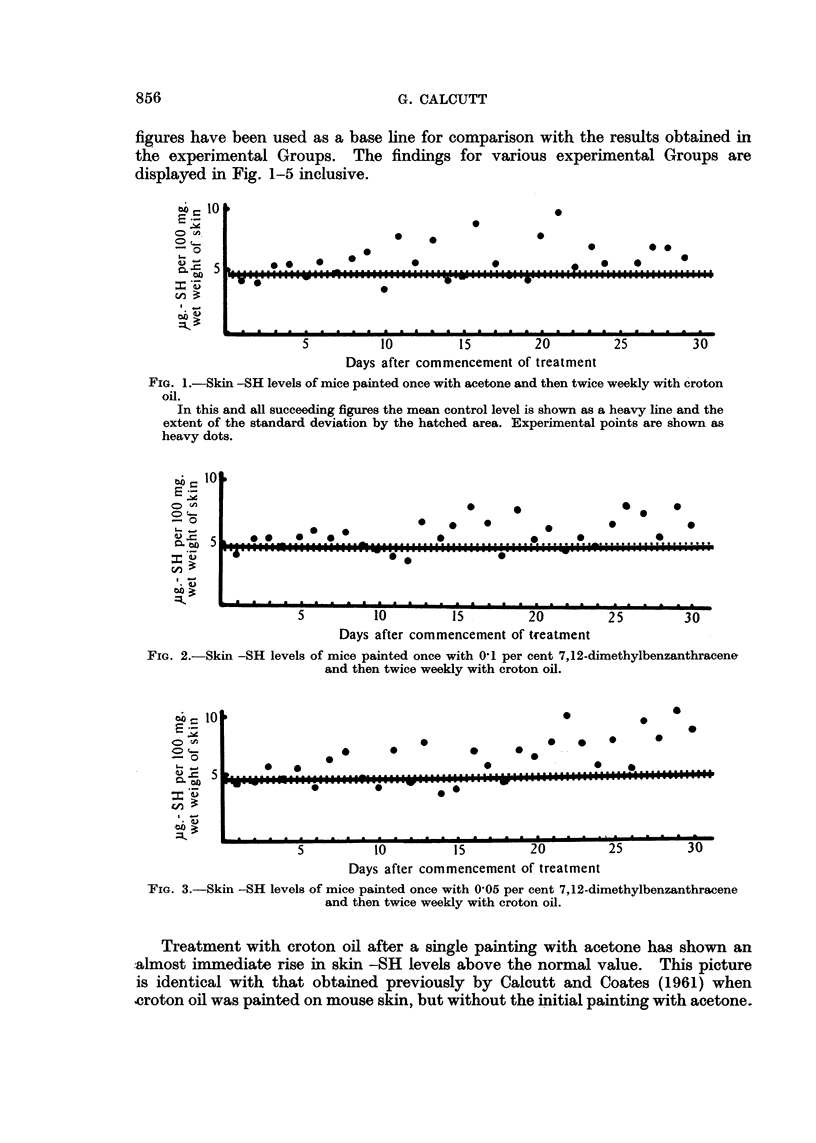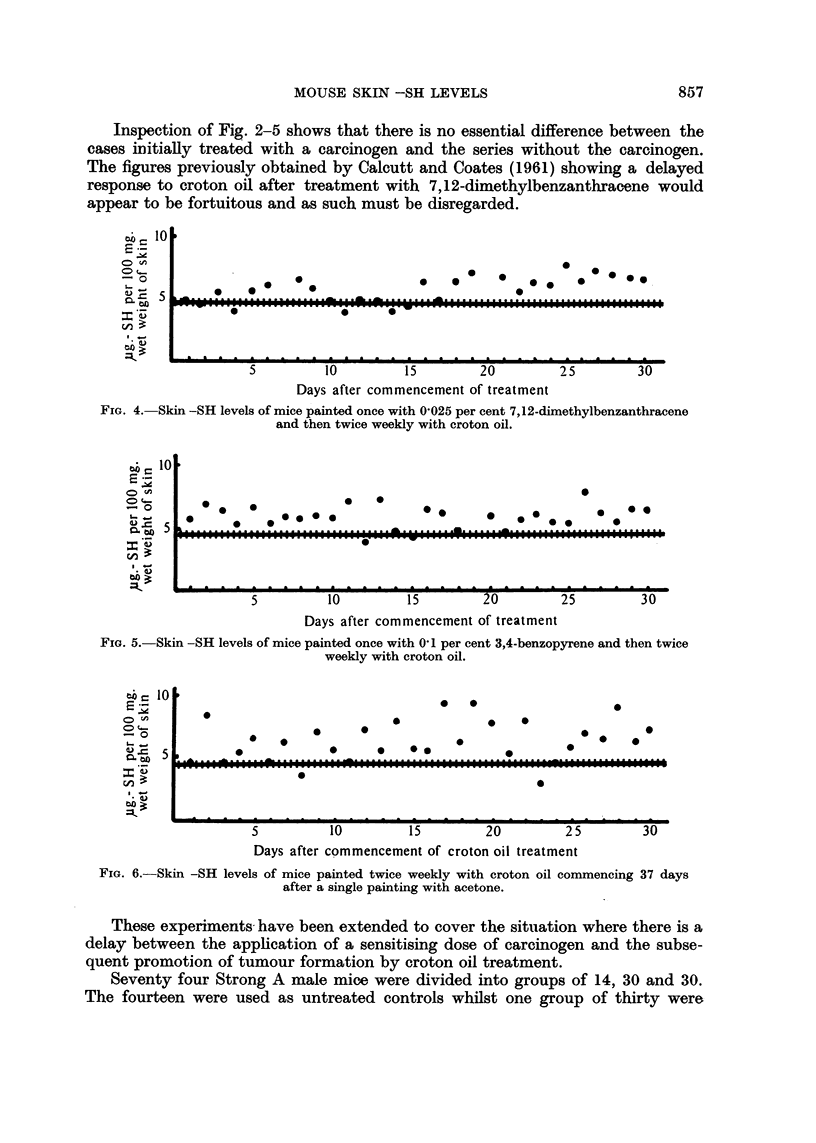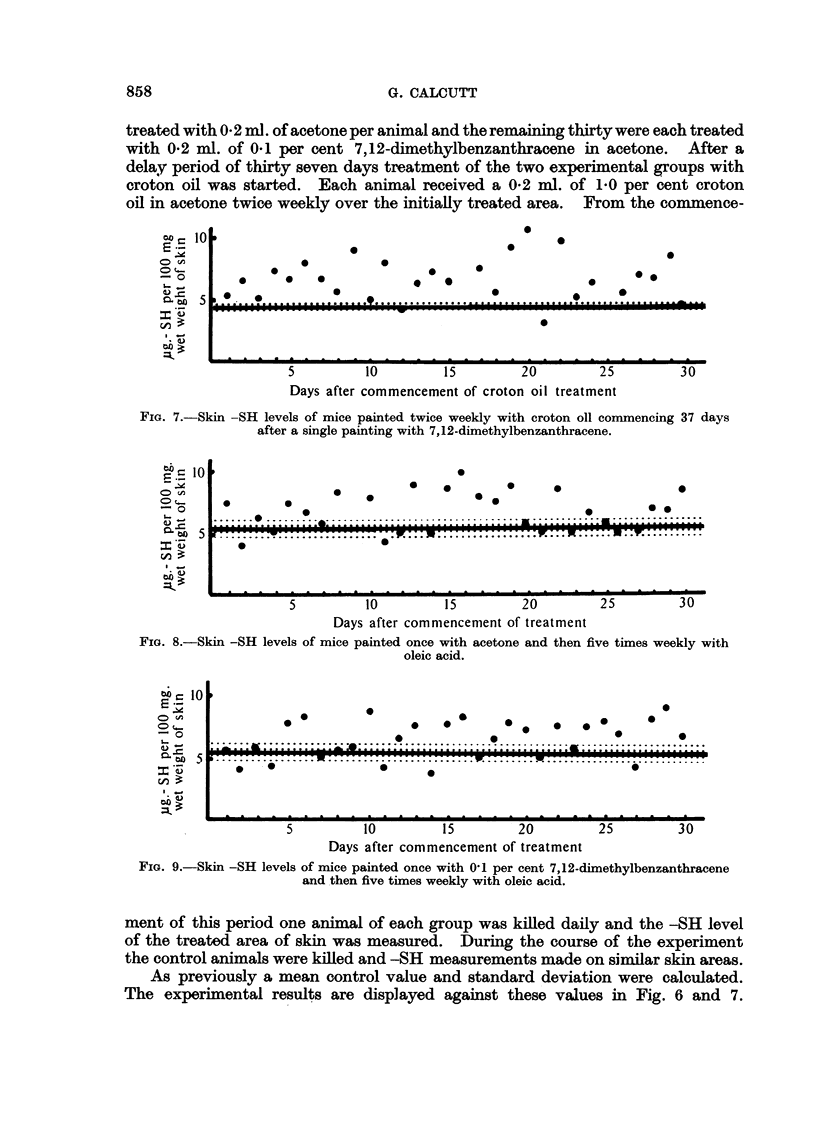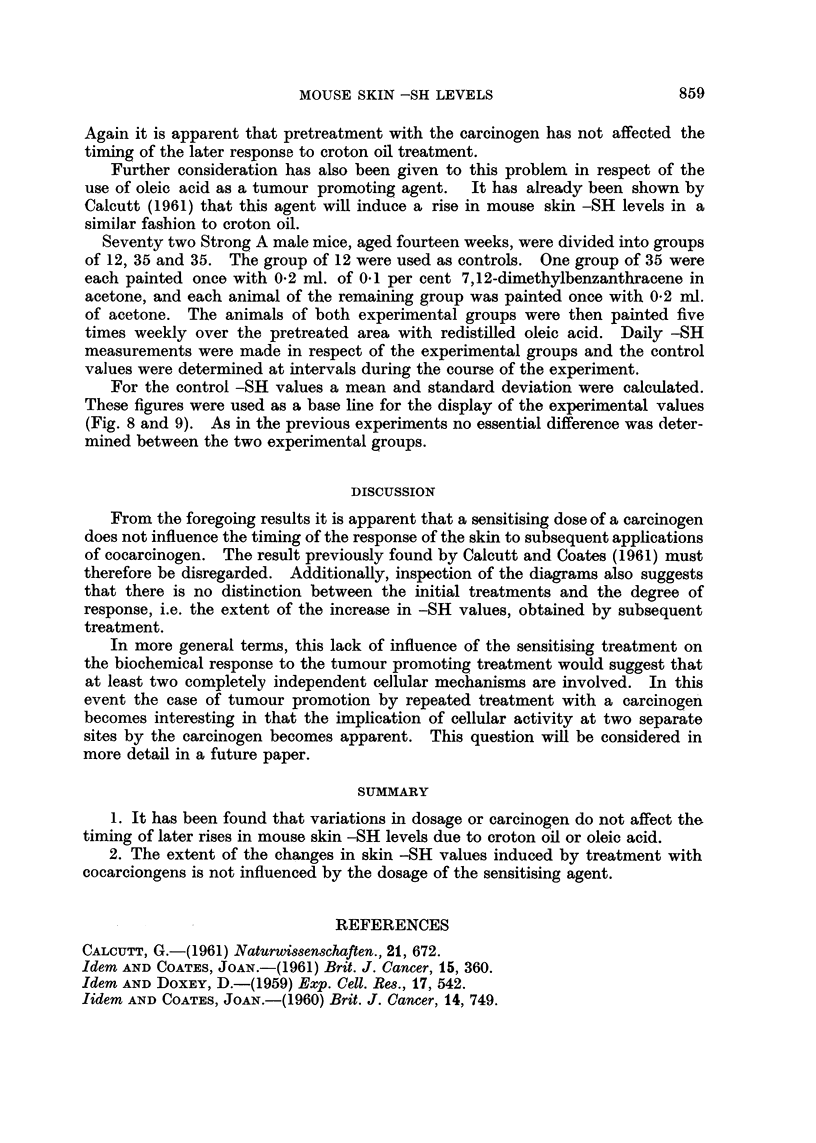# A Further Study of the Effects of Polycyclic Hydrocarbons and Croton Oil on Mouse Skin -SH Levels

**DOI:** 10.1038/bjc.1961.99

**Published:** 1961-12

**Authors:** G. Calcutt


### A FURTHER STUDY OF THE EFFECTS OF POLYCYCLIC HYDROCARBONS AND CROTON OIL ON MOUSE SKIN -SH LEVELS

G. CALCUTT

*From the Department of Cancer Research, Mount Vernon Hospital and the Radium Institute, Northwood, Middlesex*

Received for publication October 28, 1961

DURING a previous study of the effects of polycyclic hydrocarbons and croton oil on mouse skin sulphydryl (–SH) levels Calcutt and Coates (1961) found a time difference in the response to croton oil alone as compared with croton oil after a sensitising treatment with 7,12-dimethylbenzanthracene. This raised the question as to whether the sensitising dose of carcinogen had caused some tissue damage which had influenced the later response to croton oil.

The present work was designed to examine this possibility further and also to determine whether any similar effect was obtained when oleic acid was used as a promoting agent in the place of croton oil

All measurements of tissue –SH levels were made as in the previous work. Details of the technique are given by Calcutt and Doxey (1959) and Calcutt, Doxey and Coates (1960).

### EXPERIMENTAL

One hundred and sixty Strong A male mice, all twelve weeks old, were divided into groups and treated as below :—

Group I—10 mice : untreated as controls.

Group II—30 mice : painted once with 0·2 ml. of acetone.

Group III—30 mice : painted once with 0·2 ml. of 0·1 per cent 7,12-dimethylbenzanthracene in acetone.

Group IV—30 mice : painted once with 0·2 ml. of 0·05 per cent 7,12–dimethylbenzanthracene in acetone.

Group V—30 mice : painted once with 0·2 ml. of 0·025 per cent dimethylbenzanthracene in acetone.

Group VI—30 mice : painted once with 0·2 ml. of 0·1 per cent 3,4-benzopyrene in acetone.

All animals in Groups II to VI inclusive‘ were then painted twice weekly with 0·2 ml. of 1·0 per cent croton oil in acetone. The treatments in all cases were to an area of approximately 1 cm. square in the middle of the back.

At daily intervals, commencing 24 hr. after the initial treatment, one animal was taken from each of Groups II to VI inclusive, killed and the total –SH content (protein bound plus glutathione –SH) estimated in the treated area of skin. At intervals during the course of the experiment one animal from Group I was killed and the total –SH content for a comparable piece of skin was measured.

The results in respect of Group I (control) animals showed only minor fluctuations in –SH levels so a mean and standard deviation were calculated. These figures have been used as a base line for comparison with the results obtained in the experimental Groups. The findings for various experimental Groups are displayed in Fig. 1–5 inclusive.

FIG. 1.—Skin –SH levels of mice painted once with acetone and then twice weekly with croton oil.

In this and all succeeding figures the mean control level is shown as a heavy line and the extent of the standard deviation by the hatched area. Experimental points are shown as heavy dots.

FIG. 2.—Skin –SH levels of mice painted once with 0·1 per cent 7,12-dimethylbenzanthracene and then twice weekly with croton oil.

FIG. 3.—Skin –SH levels of mice painted once with 0·05 per cent 7,12-dimethylbenzanthracene and then twice weekly with croton oil.

Treatment with croton oil after a single painting with acetone has shown an almost immediate rise in skin –SH levels above the normal value. This picture is identical with that obtained previously by Calcutt and Coates (1961) when croton oil was painted on mouse skin, but without the initial painting with acetone.

Inspection of Fig. 2–5 shows that there is no essential difference between the cases initially treated with a carcinogen and the series without the carcinogen. The figures previously obtained by Calcutt and Coates (1961) showing a delayed response to croton oil after treatment with 7,12-dimethylbenzanthracene would appear to be fortuitous and as such must be disregarded.

FIG. 4.—Skin –SH levels of mice painted once with 0·025 per cent 7,12-dimethylbenzanthracene and then twice weekly with croton oil.

FIG. 5.—Skin –SH levels of mice painted once with 0·1 per cent 3,4-benzopyrene and then twice weekly with croton oil.

FIG. 6.—Skin –SH levels of mice painted twice weekly with croton oil commencing 37 days after a single painting with acetone.

These experiments have been extended to cover the situation where there is a delay between the application of a sensitising dose of carcinogen and the subsequent promotion of tumour formation by croton oil treatment.

Seventy four Strong A male mice were divided into groups of 14, 30 and 30. The fourteen were used as untreated controls whilst one group of thirty were treated with 0·2 ml. of acetone per animal and the remaining thirty were each treated with 0·2 ml. of 0·1 per cent 7,12-dimethylbenzanthracene in acetone. After a delay period of thirty seven days treatment of the two experimental groups with croton oil was started. Each animal received a 0·2 ml. of 1·0 per cent croton oil in acetone twice weekly over the initially treated area. From the commence-

FIG. 7.—Skin –SH levels of mice painted twice weekly with croton oll commencing 37 days after a single painting with 7,12-dimethylbenzanthracene.

FIG. 8.—Skin –SH levels of mice painted once with acetone and then five times weekly with oleic acid.

FIG. 9.—Skin –SH levels of mice painted once with 0·1 per cent 7,12-dimethylbenzanthracene and then five times weekly with oleic acid.

ment of this period one animal of each group was killed daily and the –SH level of the treated area of skin was measured. During the course of the experiment the control animals were killed and –SH measurements made on similar skin areas.

As previously a mean control value and standard deviation were calculated. The experimental results are displayed against these values in Fig. 6 and 7.

Again it is apparent that pretreatment with the carcinogen has not affected the timing of the later response to croton oil treatment.

Further consideration has also been given to this problem in respect of the use of oleic acid as a tumour promoting agent. It has already been shown by Calcutt (1961) that this agent will induce a rise in mouse skin –SH levels in a similar fashion to croton oil.

Seventy two Strong A male mice, aged fourteen weeks, were divided into groups of 12, 35 and 35. The group of 12 were used as controls. One group of 35 were each painted once with 0·2 ml. of 0·1 per cent 7,12-dimethylbenzanthracene in acetone, and each animal of the remaining group was painted once with 0·2 ml. of acetone. The animals of both experimental groups were then painted five times weekly over the pretreated area with redistilled oleic acid. Daily –SH measurements were made in respect of the experimental groups and the control values were determined at intervals during the course of the experiment.

For the control –SH values a mean and standard deviation were calculated. These figures were used as a base line for the display of the experimental values (Fig. 8 and 9). As in the previous experiments no essential difference was determined between the two experimental groups.

### DISCUSSION

From the foregoing results it is apparent that a sensitising dose of a carcinogen does not influence the timing of the response of the skin to subsequent applications of cocarcinogen. The result previously found by Calcutt and Coates (1961) must therefore be disregarded. Additionally, inspection of the diagrams also suggests that there is no distinction between the initial treatments and the degree of response, i.e. the extent of the increase in –SH values, obtained by subsequent treatment.

In more general terms, this lack of influence of the sensitising treatment on the biochemical response to the tumour promoting treatment would suggest that at least two completely independent cellular mechanisms are involved. In this event the case of tumour promotion by repeated treatment with a carcinogen becomes interesting in that the implication of cellular activity at two separate sites by the carcinogen becomes apparent. This question will be considered in more detail in a future paper.

### SUMMARY

1. It has been found that variations in dosage or carcinogen do not affect the timing of later rises in mouse skin –SH levels due to croton oil or oleic acid.

2. The extent of the changes in skin –SH values induced by treatment with cocarciongens is not influenced by the dosage of the sensitising agent.

### REFERENCES

CALCUTT, G.—(1961) *Naturwissenschaften.*, **21**, 672.
*Idem* AND COATES, JOAN.—(1961) *Brit. J. Cancer*, **15**, 360.
*Idem* AND DOXEY, D.—(1959) *Exp. Cell. Res.*, **17**, 542.
*Iidem* AND COATES, JOAN.—(1960) *Brit. J. Cancer*, **14**, 749.